# The Head Quarters of Drunkenness

**Published:** 1866-12

**Authors:** 


THE HEAD QUARTERS OF DRUNKENNESS.—Liverpool has been pronounced the most drunken town in England. And it is true. Its extreme drunkenness arrests the attention of the judges, its pauperism weighs heavily upon the rate-payers; its rate, fifty-six per thousand, appalling. The drunken cases dealt summarily with by the magistrates are set down at the annual rate of one in thirty-three of the population. The habitual drunkards, in their periodic appearances before the bench, form an endless chain of besotted creatures. According to the recently published judicial statistics there are 3,100 habitual drunkards in Liverpool, and they are about equally divided as to sexes.—[Liverpool Albion.

